# Biosynthesized Iron Oxide Nanoparticles (Fe_3_O_4_ NPs) Mitigate Arsenic Toxicity in Rice Seedlings

**DOI:** 10.3390/toxics9010002

**Published:** 2020-12-31

**Authors:** Sehresh Khan, Nazneen Akhtar, Shafiq Ur Rehman, Shaukat Shujah, Eui Shik Rha, Muhammad Jamil

**Affiliations:** 1 Department of Biotechnology and Genetic Engineering, Kohat University of Science & Technology (KUST), Kohat 26000, Pakistan; sehreshkhan91@yahoo.com (S.K.); nazneen_kht92@yahoo.com (N.A.); 2 Department of Biology, University of Haripur, Haripur 22620, Pakistan; drshafiq@yahoo.com; 3 Department of Chemistry, Kohat University of Science & Technology (KUST), Kohat 26000, Pakistan; shaukat.shujah@yahoo.com; 4 Department of Well-being Resources, Sunchon National University, Suncheon 540-742, Korea

**Keywords:** *Bacillus subtilis*, iron oxide nanoparticles, rice plant, UV, XRD, FTIR, SEM

## Abstract

Arsenic (As) contamination has emerged as a serious public health concern worldwide because of its accumulation and mobility through the food chain. Therefore, the current study was planned to check the effect of *Bacillus subtilis*-synthesized iron oxide nano particles (Fe_3_O_4_ NP) on rice (*Oryza Sativa* L.) growth against arsenic stress (0, 5, 10 and 15 ppm). Iron oxide nanoparticles were extracellular synthesized from *Bacillus subtilis* with a desired shape and size. The formations of nanoparticles were differentiated through UV-Visible Spectroscopy, FTIR, XRD and SEM. The UV-Visible spectroscopy of *Bacillus subtilis*-synthesized nanoparticles showed that the iron oxide surface plasmon band occurs at 268 nm. FTIR results revealed that different functional groups (aldehyde, alkene, alcohol and phenol) were present on the surface of nanoparticles. The SEM image showed that particles were spherical in shape with an average size of 67.28 nm. Arsenic toxicity was observed in seed germination and young seedling stage. The arsenic application significantly reduced seed germination (35%), root and shoots length (1.25 and 2.00 cm), shoot/root ratio (0.289), fresh root and shoots weight (0.205 and 0.260 g), dry root and shoots weight (6.55 and 6.75 g), dry matter percentage of shoot (12.67) and root (14.91) as compared to control. *Bacillus subtilis*-synthesized Fe_3_O_4_ NPs treatments (5 ppm) remarkably increased the germination (65%), root and shoot length (2 and 3.45 cm), shoot/root ratio (1.24) fresh root and shoot weight (0.335 and 0.275 mg), dry root and shoot weight (11.75 and 10.6 mg) and dry matter percentage of shoot (10.40) and root (18.37). Results revealed that the application of Fe_3_O_4_ NPs alleviated the arsenic stress and enhanced the plant growth. This study suggests that *Bacillus subtilus*-synthesized iron oxide nanoparticles can be used as nano-adsorbents in reducing arsenic toxicity in rice plants.

## 1. Introduction

Arsenic (As)-polluted water is a serious problem in Pakistan [1], which is further aggravated by industrialization and agricultural practices [2]. It enters in the human body through inhalation, ingestion or skin absorption and affects different organs [3]. Rice is an essential staple food grown under flooded conditions; if irrigated with As-contaminated water, the same accumulates in different parts of plant tissues and is subsequently transported to the human food chain [4]. Presently, various methods, such as physical, chemical and biological methods, have been practiced in order to remediate As from water. Physical and chemical techniques are not suitable in the long run due to being costly, having lower efficacy and being less environmentally friendly [5], whereas, in biologically synthesized nanoparticles, different “functional groups” are naturally present on nanoparticle surfaces and heavy metals can get adsorbed on these functional groups [6]. Nanotechnology has offered a pervasive solution to these conventional technologies in the form of nanoparticles such as iron oxide nanoparticles (Fe_3_O_4_ NPs), which are more convenient for the removal of arsenic from water because of their important features like magnetic and electric properties [7]. Fe_3_O_4_ NPs have high fabrication and adsorption capacities for arsenic and super-paramagnetic properties which allows easy separation from water and makes them popular candidates for remediation of arsenic-contaminated water.

Biological routes for synthesizing metal nanoparticles through microbes are gaining much attention due to their low toxicity, bio-compatibility and environmentally friendly nature. Different bacterial species such as *Actinobacter* sp., *Actinobacter* sp., *Thermoanaerobacter* sp., *Bacillus subtilis* and *Thiobacillus thioparus* were used for synthesis of Fe_3_O_4_ nanoparticles [8]. According to Ahmad [9], microbes are considered as a potential hub for nanoparticle synthesis. Synthesis of nanoparticles by using bacteria is an emerging and upcoming research because bacteria such as *Bacillus subtilis* contains extracellular enzymes that act as an electron shuttle in reducing metal. They also release reducing agents like hydroquinones, which are capable of reducing metal ions to nanoparticles [6]. Positive impacts of Fe_3_O_4_ NPs have been observed on plant growth because it could be used as an iron fertilizer at low concentration (5–20 ppm) and alleviate arsenic induced toxicity [10,11,12].

Chemically synthesized Fe_3_O_4_ NPs have been used for remediation of arsenic-contaminated water but no study so far has been reported on the effect of biologically “*Bacillus subtilis”*-synthesized Fe_3_O_4_ NPs on remediation of arsenic-contaminated water. Therefore, the present study was designed to synthesize Fe_3_O_4_ NPs from *Bacillus subtilis* and investigate their remediating effect on rice (*Oryza sativa* L.) growth in arsenic-polluted water. Furthermore, the impact of *Bacillus subtilis*-synthesized Fe_3_O_4_ NPs on rice plant growth has also been studied.

## 2. Materials and Methods

### 2.1. Experimental Methods

The fresh culture of *Bacillus subtilis* and rice seed of the variety Super Basmati were taken from NARC (National Agriculture Research Center), Islamabad, Pakistan. The seeds were sterilized with a 3% solution of sodium hypochlorite for 3 minutes and washed thoroughly with distilled water [13]. The amount of seed used in each experiment was 10 and the number of replicates was 5.

*Bacillus subtilis* culture was re-fresh again and grown in a fresh Luria Broth medium at 37 °C (150 rpm) for 36 h. Bacteria culture was centrifuged at 4000 rpm for 12 minutes and the culture supernatant was used for the synthesis of iron nanoparticles. The protocol of Sundaram [6] was used for the biological synthesis of Fe_3_O_4_ NPs. An aqueous solution of 2 mM Fe_2_O_3_ (15 mL) was treated with 5 mL of *Bacillus subtilis* supernatant solution in a 250 mL Erlenmeyer flask (pH adjusted to 8.5). The flask was incubated under dark conditions along with the control (Fe_2_O_3_ and *Bacillus subtilis* extract) at 35 °C (200 rpm) for 5 days. Fe_3_O_4_ NPs were characterized by UV-Vis double beam spectrophotometer and X-ray diffraction, which is usedfor phase identification and characterization of the crystalline nature of Fe_3_O_4_ nanoparticles. The morphology of nanoparticles was determined by scanning electron microscopy SEM (HITACHI, S-3000H). FTIR (Perkin-Elmer Spectrum RXI) analysis was used for the characterization of iron oxide nanoparticles. The size of nanoparticles was calculated using Sherrer’s equation D = kƛ/Bcosθ [14].

D = mean grain size, k = geometricfactor, ƛ = X-ray wavelength, B = FWHM (Full width at half maximum) and θ is the diffraction angle.

### 2.2. Application of Bacillus subtilis-Synthesized Fe_3_O_4_ NPs on Seed Germination

Seeds were placed in Petri plates by applying different concentrations of As_2_O_3_ (5, 10 and 15 ppm) along with a Fe_3_O_4_ nanoparticle (5, 10 and 15 ppm) solution under dark conditions. The germination percentage was recorded for 5 days.

### 2.3. Determination of Physiological Parameters

After two weeks, plants were taken randomly from each treatment and were separated into roots and shoots. The root–shoot length of the plant was measured in centimeters (cm), while fresh and dry weight was measured in grams (g).

### 2.4. Experimental Design and Statistical Analysis

The experiment was designed to replicate. The physiological results are represented as mean ± SE (*n* = 5). Statistics 9 software version (v.10) (Informer technologies, Inc., Los Angeles, CA, USA) was used for statistical analysis. Duncan’s multiple range test was performed to determine the least significant difference (LSD) between treatments at a *p* < 0.05 significant level.

## 3. Results

### 3.1. Fe_3_O_4_ NPs Synthesis and Characterization

**Figure 1:**
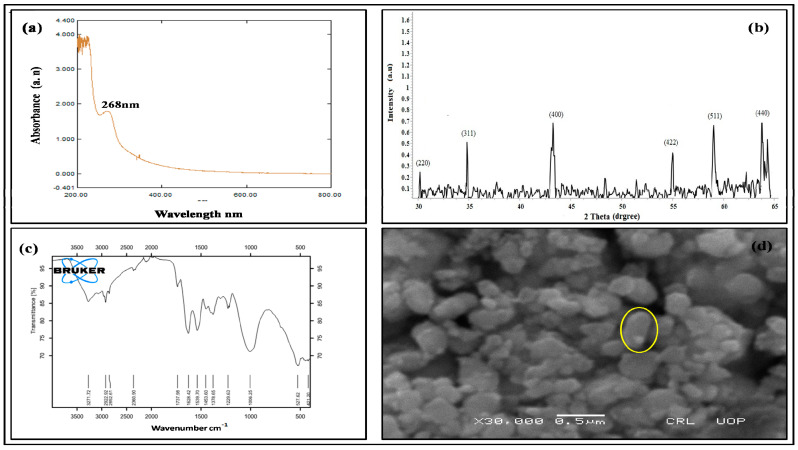
Representative UV-Vis absorption spectra (**a**), XRD pattern (**b**), FTIR spectra (**c**), SEM image, 0.5 µm scale (**d**) of iron oxide nanoparticles synthesized by adding 15 mL of iron oxide suspension with 5 mL of *Bacillus subtilus* extract.

Fe_3_O_4_ NPs synthesis was done using the *Bacillus subtilis* extract because it contains reducing compounds which increase the synthesis of NPs [6]. To characterize the biologically synthesized Fe_3_O_4_ NPs, UV-Vis spectroscopy, FTIR, XRD and SEM were used (Figure 1).

UV-Vis spectrum of *Bacillus subtilis*-synthesized nanoparticles showed that the iron oxide surface plasmon band occurs at 268 nm (Figure 1a). XRD pattern of *Bacillus subtilis*-mediated iron oxide nanoparticles revealed different peaks at 2θ = 30.17°, 35.46°, 43.25°, 54.99°, 57.23° and 63.79°, respectively indexed to 220, 331, 400, 442, 511 and 440 Bragg reflection. XRD peaks of nanoparticles were compared with the Joint Committee on Powder Diffraction Standards (JCPDS). In the current study, the X-ray peak position was used to determine the Fe_3_O_4_ NPs crystallinity. The size (67.28 nm) was calculated by using the Scherrer equation and the shape was cubic spinel in structure (Figure 1b).

The FTIR analysis was performed to identify the presence of different function groups such as aldehyde, alkene, alcohol and phenolon the surface of iron oxide nanoparticles. FTIR spectra showed the interaction between iron salt (Fe_2_O_3_) and protein molecules, which revealed the reduction between iron ions and the stabilization of iron nanoparticles. The observed bands were between 527.62 and 421.20 cm^−1^ for iron oxide and 2922.92, 2852.61 and 1737.98 cm^−1^ for aldehyde due to capping of the carboxylic acid moiety. The peaks were observed in the region of 1628.42 cm^−1^ for alkene, 1006.25 cm^−1^ for alcohol and phenol (Figure 1c).

The surface morphology of the *Bacillus subtilis*-synthesized iron oxide nanoparticles was analyzed by SEM. The result of the SEM image revealed that nanoparticles were uniformly distributedas well as a few aggregates. The SEM image also showed that particles were spherical in shape with an average size of about 60–80 nm (Figure 1d).

### 3.2. Effect of Fe_3_O_4_ NPs on Seeds Germination and Growth of Seedling against Arsenic Stress

**Figure 2:**
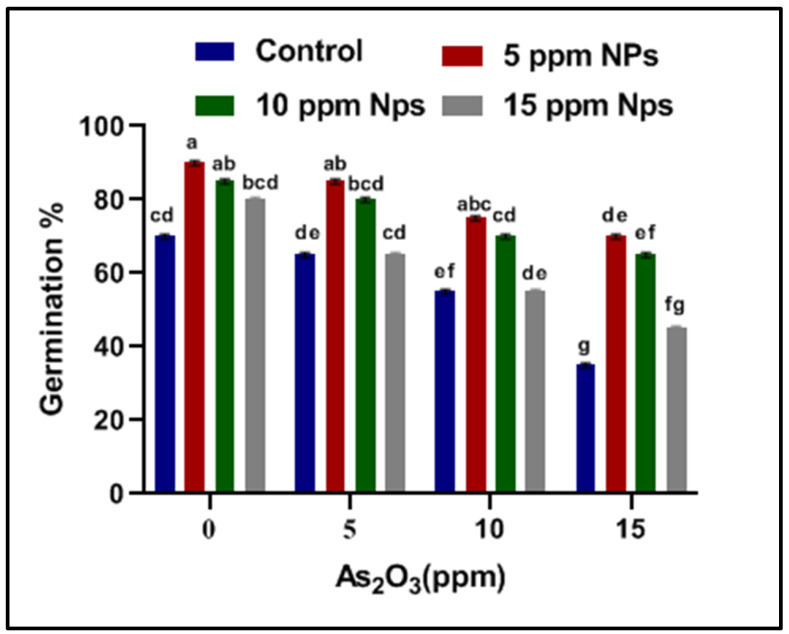
Effect of *Bacillus subtilis*-synthesized Fe_3_O_4_ NPs on “seed germination (%)” of rice (*Oryza sativa* L.) in arsenic-contaminated water. Different letters show a significant difference at *p* < 0.05 between treatment.

**Figure 3:**
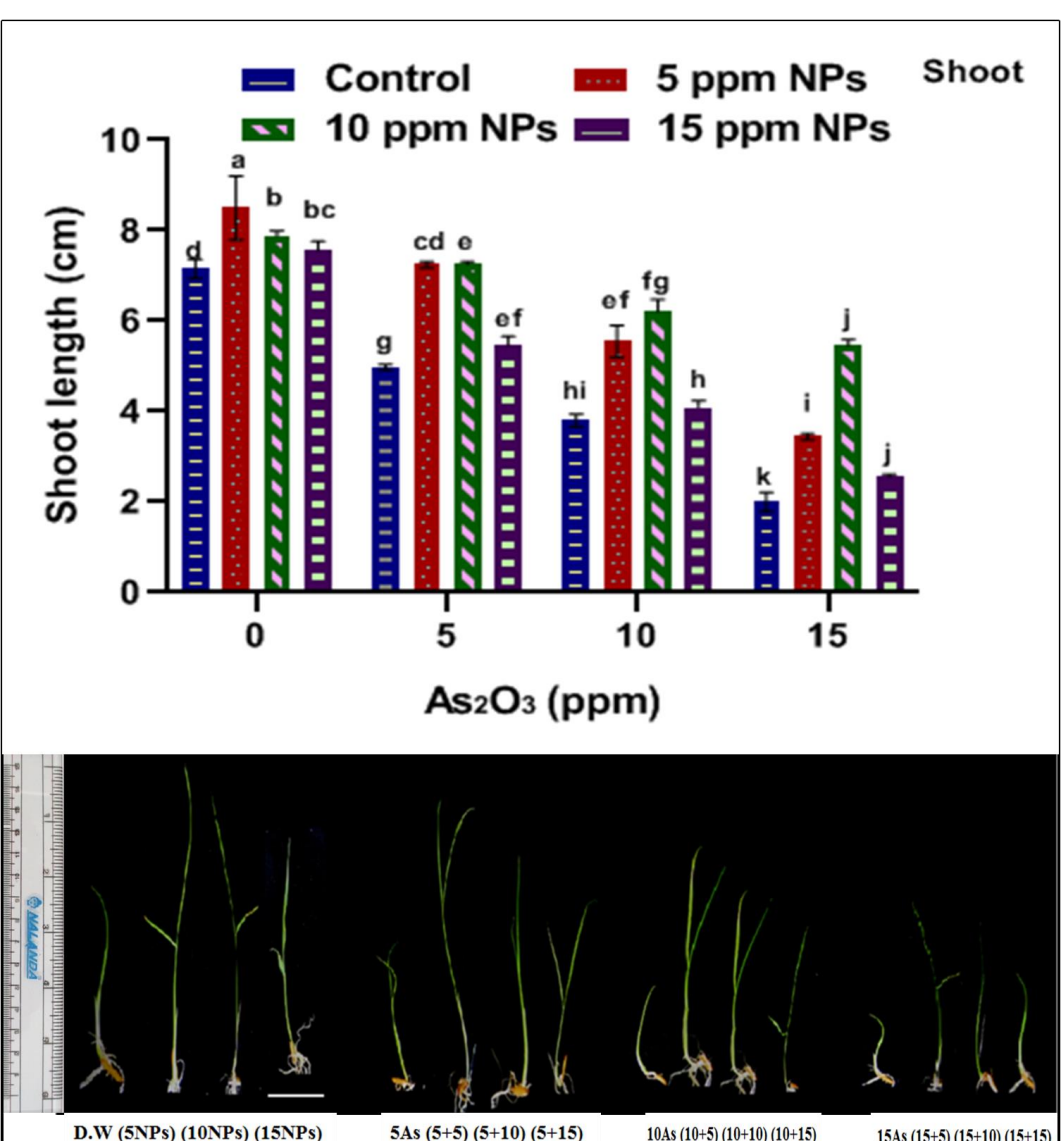
Effect of *Bacillus subtilis*-synthesized Fe_3_O_4_ NPs on shoot length of rice (*Oryza sativa* L.) in arsenic-contaminated water. Different letters show a significant difference at *p* < 0.05 between treatments.

**Figure 4:**
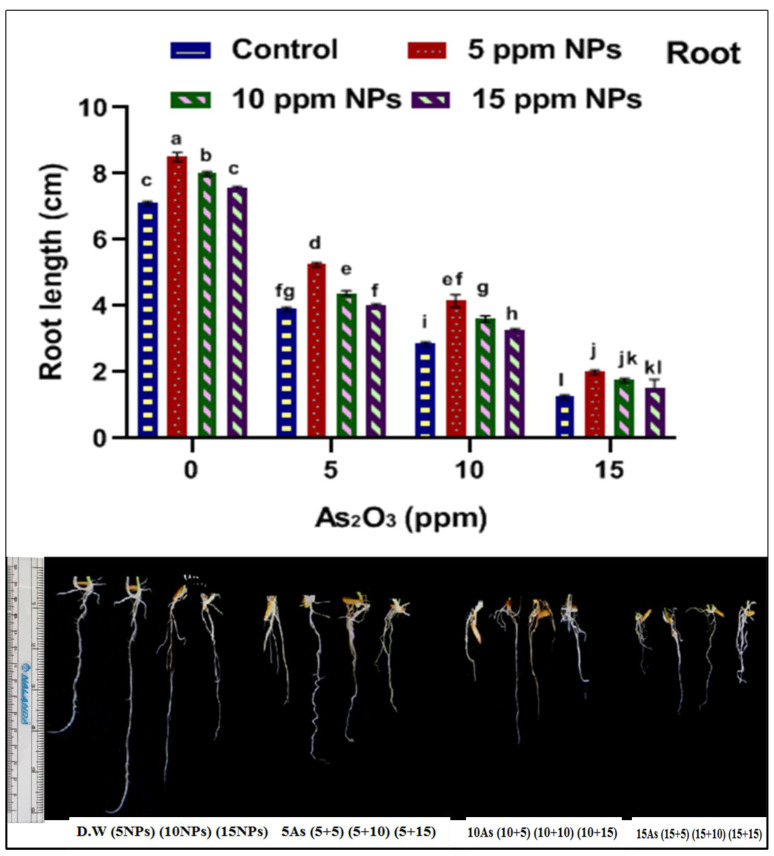
Effect of *Bacillus subtilis*-synthesized Fe_3_O_4_ NPs on root length of rice (*Oryza sativa* L.) in arsenic-contaminated water. Different letters show a significant difference at *p* < 0.05 between treatments.

**Figure 5:**
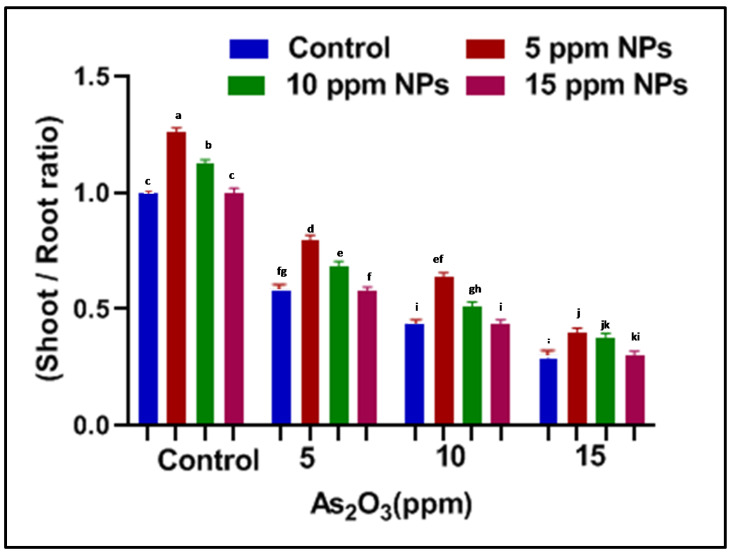
Effect of *Bacillus subtilis*-synthesized Fe_3_O_4_ NPs on shoot/root ratio of rice (*Oryza sativa* L.) in arsenic-contaminated water. Different letters show a significant difference at *p* < 0.05 between treatments.

**Figure 6:**
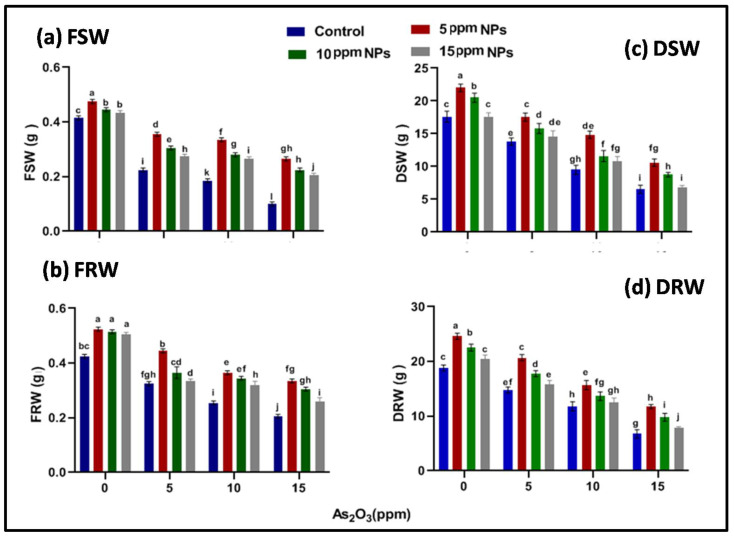
Effect of *Bacillus subtilis*-synthesized Fe_3_O_4_ NPs on (**a**) fresh shoot (**b**) fresh root weight and (**c**) dry shoot (**d**) dry root weight of rice (*Oryza sativa* L.) in arsenic-contaminated water. Different letters show a significant difference at *p* < 0.05 between treatments.

**Figure 7:**
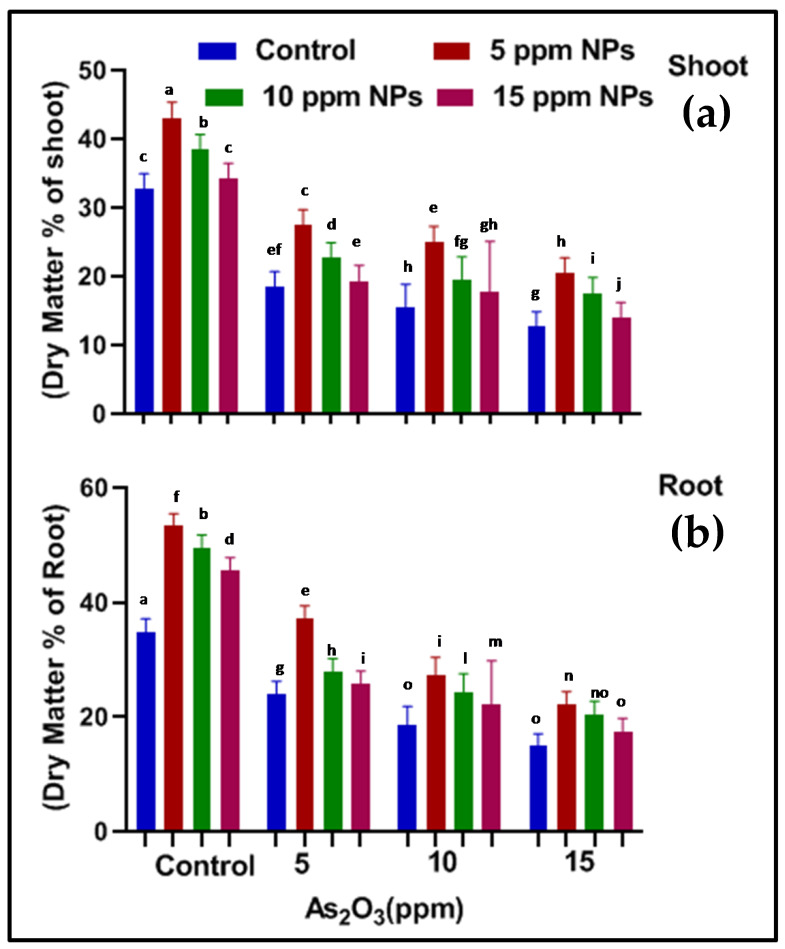
Effect of *Bacillus subtilis*-synthesized Fe_3_O_4_ NPs on (**a**) shoot (**b**) root dry matter % of rice (*Oryza sativa* L.) in arsenic-contaminated water. Different letters show a significant difference at *p* < 0.05 between treatments.

Seed germination is one of the most important parameters to assess in this study. To check the iron nanoparticles’ effect on seed germination against arsenic stress, different concentrations of Fe_3_O_4_ NPs (5, 10 and 15 ppm) solutions were applied. The impact of Fe_3_O_4_ NPs on physiological growth against arsenic application was estimated in terms of seed germination, root–shoot length and fresh–dry weight (Figure 2, Figure 3, Figure 4, Figure 5, Figure 6 and Figure 7). Among all treatments, maximum seed germination was recorded in rice plant treated with 5 ppm of Fe_3_O_4_ NPs. Application of arsenic at 15 ppm significantly reduced seed germination (35%), root–shoot length (1.25 and 2 cm), shoot/root ratio (0.289), fresh root and shoot weight (0.205 and 0.1 mg), dry root and shoot weight (6.75 and 6.55 mg) and dry biomass percentage of shoot (12.67%) and root (14.91%) in comparison to control. Thus, it was observed that seed germination, root–shoot length and fresh and dry root–shoot weight were reduced due to arsenic stress.

However, low concentrations of Fe_3_O_4_ NPs treatments showed remarkable increases in plant growth as compared with control under abiotic stress (Figure S1). Iron nanoparticles reduced the uptake of the arsenic and protected the seeds from abiotic stress by enhancing seed germination, root and shoot length, shoot/root length ratio, fresh and dry weight and dry matter percentage when compared with control. The results indicate that the different dose of iron oxide nanoparticles had a significant effect on the germination and physiological parameter of rice. Significant increases in germination (65%), root–shoot length (2 and 3.45 cm), shoot/root length ratio (1.24), fresh root and shoot weight (0.335 and 0.275 mg), dry root and shoot weight (11.75 and 10.6 mg) and dry matter percentage of shoot (10.40) and root (18.37) were recorded at 5 ppm Fe_3_O_4_ NPs when compared with control (Figure 2, Figure 3, Figure 4, Figure 5, Figure 6 and Figure 7).

## 4. Discussion

Biological syntheses of Fe_3_O_4_ NPs are more suitable when compared with chemical and physical synthesis methods because in a biological organism naturally reducing compounds are present which increase the synthesis of NPs. Fe_3_O_4_ NPs were confirmed through reduction reaction of microbial extract with Fe_2_O_3_. Sundaram [6] reported that different compounds of bacteria are involved in Fe_3_O_4_ NPs synthesis and capped the iron oxide nanoparticles. In bacteria, some extracellular enzymes show excellent redox properties; they act as an electron shuttle in the reduction of metal ion to form nanoparticles and stabilize them with a capping agent. Biologically synthesized nanoparticles are non-toxic and cost effective and biocompatible with new environments as compared to chemically synthesized iron oxide nanoparticles. Tiquia-Arashiro [15] reported that natural reducing agents like hydroquinones released by microorganisms are capable of reducing ions to nanoparticles. Capping agents like protein or secondary metabolites secreted by bacteria capped/stabilized the nanoparticles. These nanoparticles are biocompatible withnew environments.

Characterization of Fe_3_O_4_ NPs was completed using UV-Vis spectrophotometer; FTIR, XRD and SEM. The UV-Vis spectroscopy results showed that a plasmon band of biosynthesized iron oxide nanoparticles occurred at around 268 nm, suggesting that the particles are uniformly dispersed in the aqueous solution. Nanoparticles are stabilized through capping by different bacterial proteins like hydroquinone [6]. X-ray diffraction analysis, which was used to identify the size and characterization of crystalline nature of Fe_3_O_4_ nanoparticles, also confirmed that particles are uniformly dispersed in the aqueous solution. Sundaram [6] also confirmed similar results in XRD pattern that the particle was well dispersed in aqueous solution and stabilized by a capping agent like proteins secreted by *Bacillus subtilis*. The overall result of FTIR spectra indicated that different organic groups OH, C-H, C=O, C=C and C-O were present which act as a capping agent in iron oxide nanoparticles, the strong band at 527.62 and 421.20 cm^−1^ were due to specific vibrations of Fe and oxygen. Different functional groups such as aldehyde, alkene, alcohol and phenol were present on the surface of nanoparticles. The band was in the region of 527.62 and 421.20 cm^−1^ for iron oxide and 2922.92, 2852.61 and 1737.98 cm^−1^ for aldehyde due to capping of the carboxylic acid moiety. The peakswere observedin the region of1628.42 cm^−1^ for alkene, 1006.25 cm^−1^ for alcohol and phenol, while, as reported in literature [6], certain organic compounds containing O-H, C=O, C-O, N-H are present which act as a capping agent in biosynthesized iron oxide nanoparticles compared to free ferric oxide. The morphology ofnanoparticles was determined by scanning electron microscopy (SEM). The nanoparticles were not in direct contact even within the aggregates, indicating stability of nanoparticles by a capping agent. *Bacillus subtilis*-synthesized nanoparticles are extremely stable, which is likely due to capping with protein or secondary metabolites secreted by the bacteria as reported by Sundaram [6].

The effect of Fe_3_O_4_ NPs was more prominent at the seed germination stage because at a lower concentration Fe_3_O_4_ NPs acts as a nutrient for seed growth [11,16,17]. It was reported that silicon regulates the growth parameters in plants under arsenic stress [16,18]. Plant growth was inhibited by arsenic stress. Arsenic (V) is phosphate analogous; through phosphate transporters it enters the plant and uncouples the oxidative phosphorylation. Arsenic (III) reacts with thiol groups and inhibits the metabolic process. The finding of Rahman [19] elaborated that the moisture content and nutrient uptake in seeds were reduced significantly with an increase of arsenic (As) concentration.

However, when the rice seeds were exposed to Fe_3_O_4_ NPs solution, it was observed that there was a significant increase in germination. Though the Fe_3_O_4_ NPs diffuses through nano-holes on the seed coat, it increases the water uptake, amylase activity and starch metabolism, which significantly improve the seed germination in green gram plants [20,21]. The surface of iron oxide nanoparticles attracts the negative charge arsenic ions and adsorb it. Adsorption of arsenic on Fe_3_O_4_ NP surfaces depends on two steps. First, arsenic ions migrate from the bulk fluid phase to the outer nanoparticle surface for contact. Second, the electrostatic attraction between adsorbate (As) and adsorbent (Fe_3_O_4_ NPs) and this complex restrict the entry of arsenic in a rice plant [22]. According to Kalita [23], roots are the initial tissue to be exposed to arsenic stress; as a result, arsenic inhibits the extension and proliferation of roots. The shoot length decreased due to inhibition of cell division and decline in the activity of hydrolytic enzyme and food not reaching the radical and plumule [24]. According to a report by Du, L. et al. [25], under arsenic stress, asignificant decrease in leaf and root biomass formation takes place. In general, growth reduction may be linked to a loss of cellular turgor, resulting in either an inhibition of cell elongation or a decrease of mitotic activity. Our finding is related to a previous study by Liu [26], which found that iron oxide nanoparticles at lower concentration are not only less toxic but also enhance the growth of lettuce seedlings and are effective as a nanofertilizer. According to the findings of Mahakham [27], Fe_3_O_4_ NPs increase plant growth by regulating gibberellin and cytokinin, which are directly involved in cell division and elongation and in reducing ethylene production. All the growth parameters are suppressed under arsenic stress because changes occur at the cellular level [28].

The current study revealed that Fe_3_O_4_ NPs treatments enhanced the growth of the rice plant when compared with control. There is very little data available on the effects of Fe_3_O_4_ NPs treatments on rice plants under arsenic stress [29]. A similar kind of result was also observed by other researchers [30,31,32,33] in rice plants in response to Fe_3_O_4_ NPs.

## 5. Conclusions

In this study, we examined the effects of Fe_3_O_4_ NPs on rice plants in response to arsenic stress. A lower concentration of Fe_3_O_4_ NPs inhibits the arsenic level significantly and enhances plant growth, whereas a higher concentration fails to do so. It seemed that Fe_2_O_3_ NPs have different effects due to their dosage. To our best understanding, this is the first report thatshows the positive effect of *Bacillus subtilis*-synthesized Fe_3_O_4_ NPs on the growth of rice plants and the capability to cope with arsenic stress. Further research is needed to identify the basic intracellular mechanism of *Bacillus subtilis* to synthesized Fe_3_O_4_ NPs and subsequently increase their efficiency to remediate arsenic-contaminated water. It is highly recommended to develop and subsequently commercialize *Bacillus subtilis-*synthesized Fe_3_O_4_ NPs.

## Data Availability

Not applicable.

## Supplementary Materials

The following are available online at https://www.mdpi.com/2305-6304/9/1/2/s1, Figure S1: Application of low concentrations of Fe_3_O_4_ NPs treatments showed remarkable increases in plant growth as compared with arsenic contaminated water.
