# Supplementary material for: Biosynthesized Iron Oxide Nanoparticles (Fe3O4 NPs) Mitigate Arsenic Toxicity in Rice Seedlings
Source: Toxics. 2020 Dec 31;9(1):2. doi: 10.3390/toxics9010002 (PMC7823513; doi:10.3390/toxics9010002)
Supplement: Supplementary file 1 [file toxics-09-00002-s001.pdf]

Supplementary Materials: Biosynthesized Iron Oxide Nanoparticles (Fe_3O_4NPs)Mitigate Arsenic Toxicity in Rice Seedlings

Sehresh Khan, Nazneen Akhtar, Shafiq Ur Rehman, Shaukat Shujah, Eui Shik Rha and Muhammad Jamil

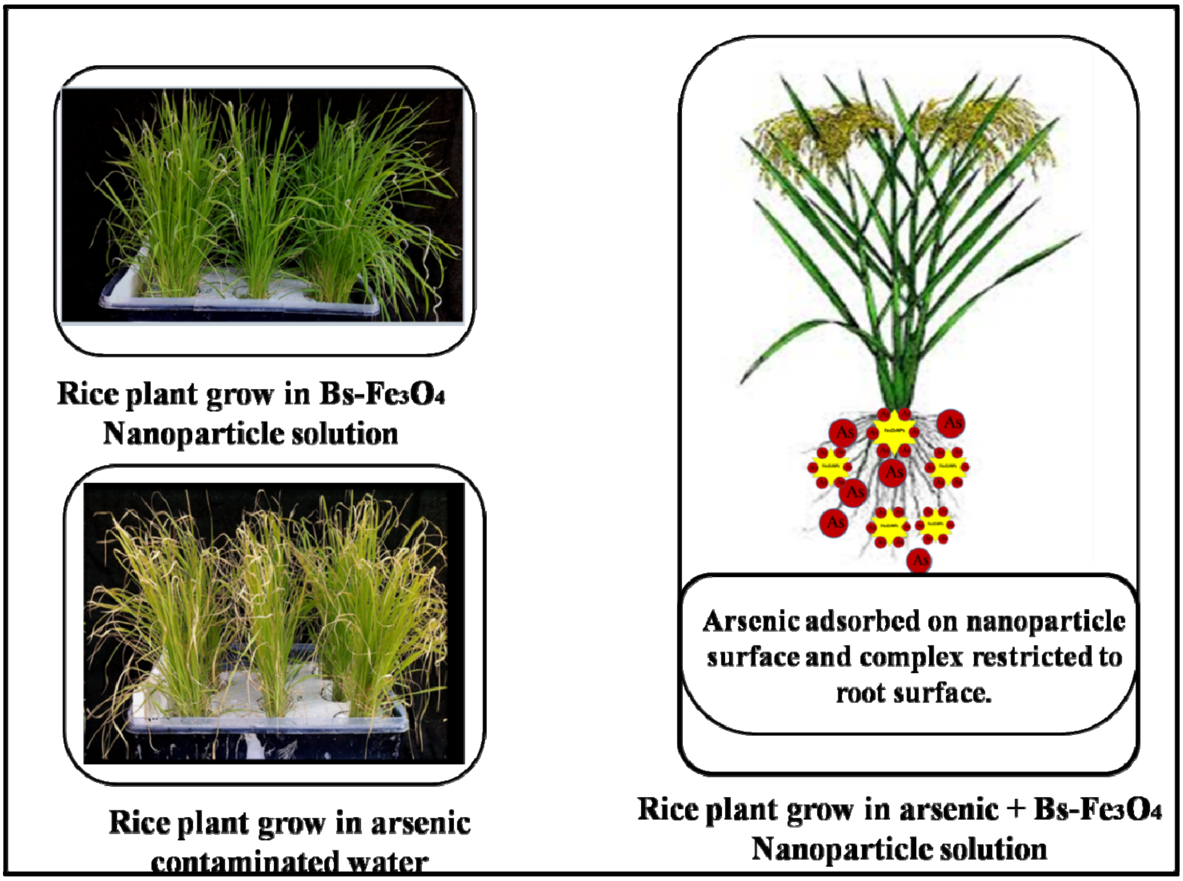

Figure S1. Application of low concentrations of Fe_3O_4 NPs treatments showed remarkable increases in plant growth as compared with arsenic contaminated water.
